# Glucagon‐Like Peptide‐1 Receptor Agonists and Major Adverse Cardiovascular Events in Patients With and Without Diabetes: A Meta‐Analysis of Randomized‐Controlled Trials

**DOI:** 10.1002/clc.24314

**Published:** 2024-07-02

**Authors:** Alireza Hosseinpour, Aayushi Sood, Jahangir Kamalpour, Ehsan Zandi, SeyedAbbas Pakmehr, Hamidreza Hosseinpour, Akshit Sood, Ankit Agrawal, Rahul Gupta

**Affiliations:** ^1^ School of Medicine Shiraz University of Medical Sciences Shiraz Iran; ^2^ Department of Medicine The Wright Center for Graduate Medical Education Scranton Pennsylvania USA; ^3^ Department of Medicine Navjivan General and Maternity Hospital Jalandhar Punjab India; ^4^ Department of Hospital Medicine Cleveland Clinic Cleveland Ohio USA; ^5^ Lehigh Valley Heart Institute, Lehigh Valley Health Network Allentown Pennsylvania USA

**Keywords:** GLP‐1, glucagon‐like peptide‐1, major adverse cardiovascular events, meta‐analysis

## Abstract

**Introduction:**

Glucagon‐like peptide‐1 receptor agonists (GLP‐1 RAs) have shown encouraging results regarding cardiovascular outcomes mainly in patients with diabetes. In the present study, we compared the efficacy of GLP‐1 RAs in cardiovascular events between patients with and without diabetes.

**Methods:**

After finding eligible studies assessing the impact of GLP‐1 RAs on cardiovascular events in patients with and without diabetes using a systematic search, we performed a meta‐analysis on randomized‐controlled trials (RCTs) comparing cardiovascular outcomes between patients taking GLP‐1 RAs and placebo stratified by the presence or absence of diabetes. Relative risk (RR) and its 95% confidence interval (CI) were set as the reporting effect size using the random‐effects model.

**Results:**

A total of 24 RCTs (50 033 with GLP‐1 RAs and 44 514 with placebo) were included. Patients on GLP‐1 RAs had lower risk of major adverse cardiovascular events (MACE) (RR 0.87, 95% CI 0.82−0.93), cardiovascular death (RR 0.88, 95% CI 0.82−0.94), myocardial infarction (MI) (RR 0.87, 95% CI 0.77−0.97), stroke (RR 0.86, 95% CI 0.80−0.92), and hospitalization for heart failure (RR 0.90, 95% CI 0.83−0.98). Both subgroups were shown to be effective in terms of MACE and mortality. Nondiabetic patients had decreased risk of hospitalization for heart failure and MI, whereas the diabetic subgroup had marginally nonsignificant efficacy.

**Conclusion:**

The findings of this meta‐analysis indicated that patients who are overweight/obese but do not have diabetes have a comparable reduction in the risk of adverse cardiovascular events as those with diabetes. These results need to be confirmed further by large‐scale randomized trials in the future.

## Graphical abstract

A meta‐analysis of 24 randomized‐controlled trials on diabetic patients or overweight/obese patients without diabetes demonstrated that taking GLP‐1 RAs can reduce the relative risk of major adverse cardiovascular events. GLP‐1 RAs are efficient in both subgroups of diabetic and nondiabetic patients.

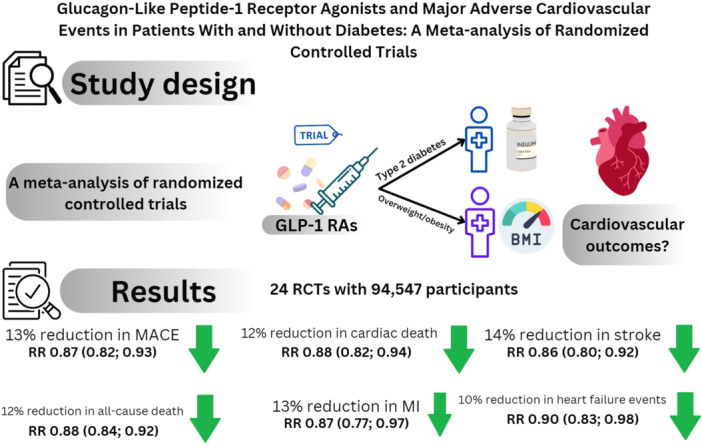

## 1 Introduction

Glucagon‐like peptide‐1 receptor agonists (GLP‐1 RAs) are a class of medications that have shown efficacy in lowering blood glucose levels and are commonly used in the treatment of type 2 diabetes [1]. GLP‐1 RAs directly stimulate the GLP‐1 receptors, leading to a strong antihyperglycemic effect by enhancing insulin secretion [2]. In the past few years, these drugs have shown promise in many areas, including their role in the reduction of cardiovascular events [3]. In 2008, the United States Food and Drug Administration issued a mandate requiring novel antihyperglycemic medications to demonstrate cardiovascular safety and efficacy through large cardiovascular outcome trials (CVOTs) [4]. Improvement in HbA1c is anticipated to reduce cardiovascular risk in diabetic patients. The cardiovascular benefits of GLP‐1 RAs demonstrated in CVOTs could be partly due to better glycemic control in patients with type 2 diabetes [5]. Although previous studies have shown the superiority of GLP‐1 RAs compared with placebo in terms of major adverse cardiovascular events (MACEs), mortality, and stroke [6, 7], there is a paucity of data on cardiovascular efficacy of GLP‐1 RAs in patients without diabetes. The recently published results of the Semaglutide Effects on Cardiovascular Outcomes in People with Overweight or Obesity (SELECT) trial, a large‐scale trial on the cardiovascular outcomes of nondiabetic overweight/obese patients taking once‐weekly injections of semaglutide compared to placebo, showed cardioprotective effects of this class of medications [8]. The majority of the previous trials have focused on the safety and efficacy of GLP‐1 RAs in diabetic patients and a considerable proportion of these patients are usually overweight/obese as well; the effects of GLP‐1 RAs on nondiabetic patients have been less commonly studied. It is noteworthy that trials of GLP‐1 RAs on nondiabetics are typically conducted on individuals who are overweight/obese. In this regard, a meta‐analysis on the cardiovascular outcomes of GLP‐1 RAs can compare these endpoints between diabetics and nondiabetics, whereas it is not possible to differentiate between overweight/obese and lean individuals as not many studies have included patients who are not overweight/obese. It is not elucidated if the cardiovascular benefits of GLP‐1 RAs have a similar magnitude of effect in patients with and without diabetes. In this meta‐analysis, we sought to compare the MACEs between patients taking GLP‐1 RAs and placebo in addition to the standard of care regarding their condition stratified by the presence or absence of diabetes.

## 2 Methods

### 2.1 Searching Process and Selection Criteria

The protocol for this systematic review was registered at PROSPERO with a registration ID of CRD42024502652. This meta‐analysis was carried out on the basis of the framework proposed by PRISMA guidelines [9]. After defining the pre‐specified inclusion and exclusion criteria, the process was started by searching specific keywords relevant to the topic in the online databases (PubMed, Scopus, and Embase). No filter was used for the search results and the time frame was from the date of inception up until November 11, 2023. The search results were updated on April 9, 2024. The combination of the following keywords was searched: ((semaglutide) OR (efpeglenatide) OR (albiglutide) OR (dulaglutide) OR (exenatide) OR (liraglutide) OR (lixisenatide) OR (GLP‐1) OR (glucagon‐like peptide 1)) AND ((cardiovascular outcome*) OR (death) OR (mortality) OR (myocardial infarction) OR (major adverse cardiovascular event*) OR (embolism) OR (thrombosis) OR (atrial fibrillation) OR (heart failure) OR (hospitalization) OR (stroke)) AND ((randomized) OR (randomised) OR (trial)) NOT (review). Next, the search results were merged into one main file, where the detection and removal of duplicates were performed. The remaining records were uploaded to the Rayyan web application [10], an online tool that aids the process of screening, and titles and abstracts were assessed in detail by two investigators (A.H. and J.K.) to find potentially eligible references. Full texts of the potentially eligible records were retrieved and assessed by reviewers based on the inclusion and exclusion criteria. Further assessment of the included articles with similar meta‐analyses was performed for additional records.

We considered the studies to be eligible based on the following inclusion criteria: (1) randomized‐controlled trials; (2) studies assessed MACEs; (3) studies compared an experimental group receiving an agent belonging to GLP‐1 agonists irrespective of the dosing regimens and route of administration; (4) studies included patients with diabetes or other conditions such as overweight/obesity and heart failure; and (5) studies with a minimum trial duration of 52 weeks (1 year). Studies were excluded if (1) outcomes compared between groups were not among cardiovascular‐related outcomes; (2) conference abstracts and lab studies; (3) the comparator arm received any active treatment including insulin, oral hypoglycemic medications, and any other agent belonging to the group of GLP‐1 agonists that the intervention arm was not receiving; (4) post hoc analysis of randomized trials; and (5) studies on glucose‐dependent insulinotropic polypeptide (GIP) and GLP‐1 dual agonists.

The primary outcome of the present study was set as MACE at the latest available follow‐up. Other outcomes of interest included cardiovascular and all‐cause death, myocardial infarction (MI), stroke/transient ischemic attack, and hospitalization for heart failure.

### 2.2 Data Extraction and Quality Appraisal

The data required for quantitative synthesis were extracted from the text, figures, and tables of the included trials. General and specific data were extracted from each article into pre‐specified spreadsheets. The general characteristics included trial name, identification number, the details of the intervention provided to the experimental group, the comorbidity of the studied population, and the duration of the longest follow‐up. The data related to the outcomes included the sample size of the intervention and comparator groups and the event rates that occurred in each of the groups.

For quality appraisal, the randomized studies included in this meta‐analysis were subjected to thorough assessment using the revised version of the Cochrane risk‐of‐bias tool for quality appraisal of randomized trials (RoB 2) [11]. This tool assesses the potential risk of bias arising from five main domains comprising randomization, deviation from the planned intervention, missing outcome data, method of measuring the endpoints of interest, and selection of the reported outcomes. Each study was assigned an overall risk of bias based on the specified criteria by two of the reviewers (J.K. and A.H.). Any disagreement was resolved by discussion and consensus. Plots visualizing the risk of bias were constructed using the robvis online tool [12].

### 2.3 Statistical Analysis

A conventional pairwise meta‐analysis was undertaken to summarize the results in R Software version 4.3.2 [13]. All the outcomes of interest were binary variables and to summarize the results, we presented a relative risk (RR) and its 95% confidence interval (CI) using “meta” and “metafor” packages. For the primary outcome (MACE), a log hazard ratio (HR) with its standard error were calculated in RevMan software using the lower and upper bounds of the reported HR. A pooled HR was calculated using a generic inverse variance method using the “metagen” function being. As a subgroup analysis, the studies were grouped by the baseline comorbidity of the studied population for all the outcomes. For each study, an RR was measured, with a weight assigned to each study based on the Mantel−Haenszel method. The variability within each subgroup with more than one study included was assessed using *I* ^2^ statistics and its associated *p*‐value. The level of heterogeneity was considered to be substantial when *I* ^2^ > 50%. As all of the results were obtained based on a subgroup analysis stratifying studies by comorbidity, assessment of publication bias by Egger's test or construction of a funnel plot was not applicable. Sensitivity analysis was also performed using the leave‐one‐out method, which excluded each of the included studies one by one and estimated the overall effect size again to determine if there was any significant change in the results. The overall results were considered statistically significant if *p* < 0.05 or if the 95% CI does not contain 1.0.

## 3 Results

### 3.1 Search Results and General Characteristics

**Figure 1:**
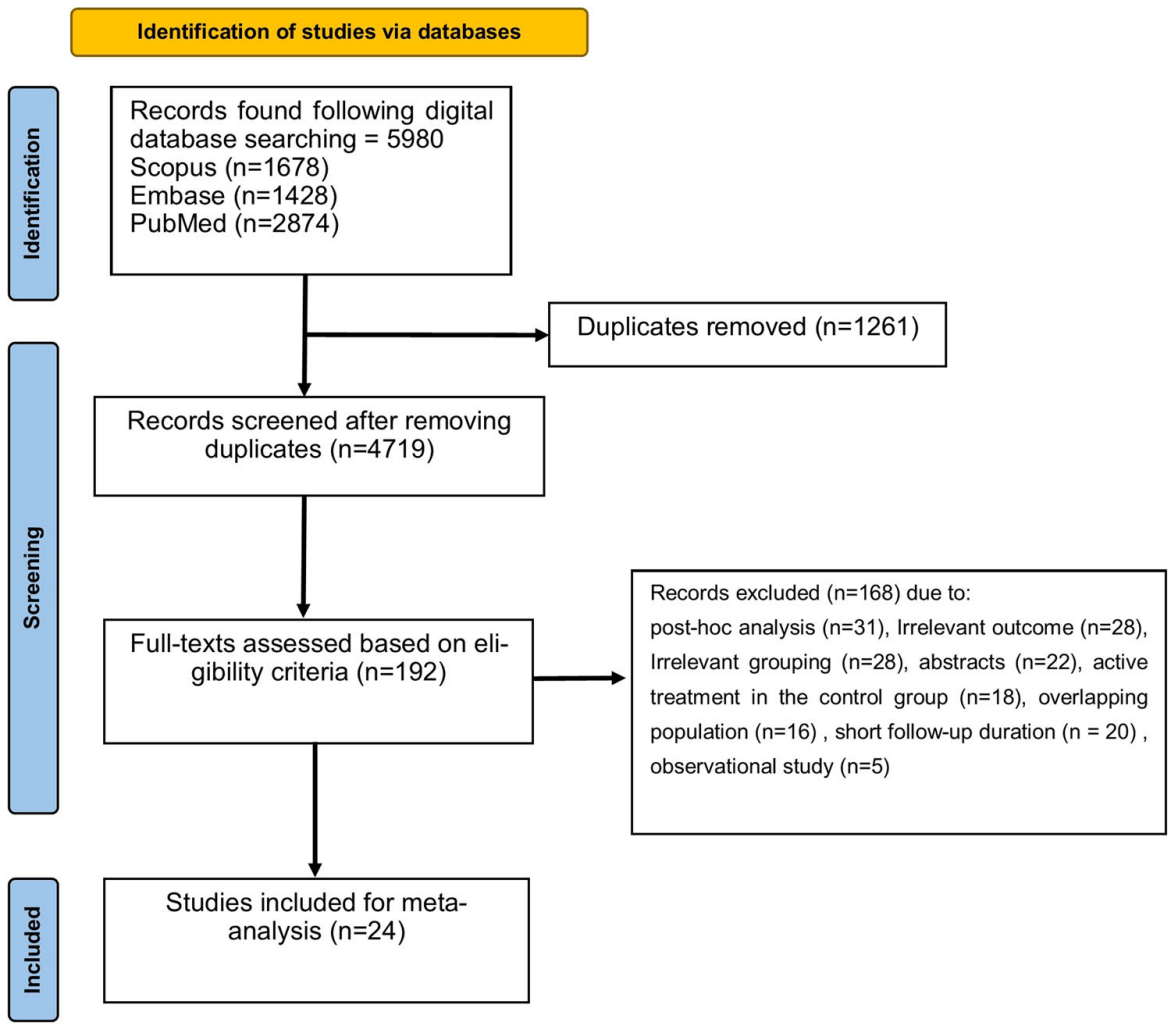
Flowchart showing the study selection process.

A total of 5980 publications were identified through a search in three digital databases, of which 1261 duplicate records were identified and then excluded. Title and abstract screening was performed for the remaining 4719 records. Then, full texts of 192 potentially eligible publications were retrieved for further assessment. Overall, a total of 24 randomized‐controlled trials comparing patients taking GLP‐1 RAs or placebo were included for quantitative synthesis [3, 8, 14, 15, 16, 17, 18, 19, 20, 21, 22, 23, 24, 25, 26, 27, 28, 29, 30, 31, 32, 33, 34, 35] (Figure 1). The total sample size was 94 547 participants (50 033 in the experimental group and 44 514 in the comparator group) and the mean (95% CI) age of the total studied population was 58.22 years (55.02; 61.43). The proportion (95% CI) of male patients was 49.56% (41.90; 57.24) among the population. The enrolled population in 17 trials included patients with type 2 diabetes mellitus [3, 14, 16, 17, 18, 19, 20, 21, 22, 23, 24, 25, 26, 27, 29, 30, 32], whereas seven studies assessed the efficacy of GLP‐1 agonists in overweight/obese patients without diabetes [8, 15, 28, 31, 33, 34, 35]. In addition to the mentioned comorbidities, patients were previously diagnosed either with a cardiovascular disease (CVD) or had high cardiovascular risk. The median follow‐up period for MACEs ranged from 1 to 5.4 years. The GLP‐1 agonists used in the experimental groups included dulaglutide, efpeglenatide, albiglutide, lixisenatide, exenatide, semaglutide, and liraglutide. These medications were administered either via the oral route or by a subcutaneous injection once a day or weekly. One study examined the efficacy of continuous subcutaneous injection of exenatide via a drug–device combination (ITCA 650) [14] (Table 1).

**Table 1:** General characteristics of the included trials.

Study	Registration ID	Intervention arm	Sample size		Inclusion criteria	Duration of follow‐up
			Experimental	Control		
REWIND	NCT01394952	Subcutaneous injection of 1.5 mg of dulaglutide weekly	4949	4952	≥50 years with T2DM and previous cardiovascular disease or high cardiovascular risk	5.4 years
AMPLITUDE‐O	NCT03496298	Subcutaneous injection of 4 or 6 mg of efpeglenatide weekly	2717	1359	T2DM with either previous cardiovascular disease or kidney disease and high cardiovascular risk	1.81 years
Harmony Outcomes	NCT02465515	Subcutaneous injection of 30−50 albiglutide weekly	4731	4732	≥40 years with T2DM and cardiovascular disease	1.5 years
EXSCEL	NCT01144338	Subcutaneous injection of 2 mg of exenatide weekly	7356	7396	T2DM with or without cardiovascular disease	3.2 years
PIONEER 6	NCT02692716	14 mg of once‐daily oral semaglutide	1591	1592	T2DM and high cardiovascular risk	15.9 months
STEP‐HFpEF	NCT04788511	Subcutaneous injection of 2.4 mg of semaglutide weekly	263	266	HFpEF and BMI ≥ 30 kg/m^2^	1 year
SELECT	NCT03574597	Subcutaneous injection of 2.4 mg of semaglutide weekly	8803	8801	≥45 years with previous cardiovascular disease and BMI ≥ 27 kg/m^2^ and no diabetes	39.8 months
LEADER	NCT01179048	Subcutaneous injection of 1.8 mg of liraglutide daily	4668	4672	T2DM and high cardiovascular risk	3.8 years
SUSTAIN‐6	NCT01720446	Subcutaneous injection of 0.5−1 mg of semaglutide weekly	1648	1649	T2DM and ≥50 years and previous cardiovascular disease or CKD stage 3 and higher or ≥60 years with high cardiovascular risk	2 years
ELIXA	NCT01147250	Subcutaneous injection of 10−20 mcg of lixisenatide daily	3034	3034	T2DM with a previous history of MI or hospitalized for unstable angina	25 months
FREEDOM CVO	NCT01455896	Continuous subcutaneous injection of ITCA 650 (20 mcg/day exenatide for 3 months, followed by 60 mcg/day for 6 months)	2075	2081	T2DM with established or at high risk of cardiovascular disease	16 months
STEP 1	NCT03548935	Subcutaneous injection of 2.4 mg (from 0.25 to 2.4 mg increased every 4 weeks) of semaglutide once a week	1306	655	BMI ≥ 30 or ≥27 kg/m^2^ with another comorbidity without diabetes	68 weeks
STEP 2	NCT03552757	Subcutaneous injection of 2.4 or 1.0 mg (from 0.25 escalated every 4 weeks) of semaglutide weekly	807	403	BMI ≥ 27 kg/m^2^ and HbA_1c_ 7%−10%	68 weeks
STEP 5	NCT03693430	Once‐weekly 2.4 mg of semaglutide	152	152	BMI ≥ 30 or ≥27 kg/m^2^ with another comorbidity without diabetes	104 weeks
HARMONY 1	NCT00849056	30 mg subcutaneous injection of albiglutide once weekly	150	151	Type 2 diabetes mellitus with BMI 20−45 kg/m^2^ on pioglitazone	3 years
HARMONY 2	NCT00849017	Once‐weekly 30 or uptitration to 50 mg of albiglutide	200	101	Type 2 diabetes and BMI 20−45 kg/m^2^ without any use of other glucose‐lowering medications	3 years
HARMONY 3	NCT00838903	Once‐weekly 30 mg subcutaneous albiglutide	302	101	Patients with type 2 diabetes and HbA_1c_ ≥ 7.0% on metformin ≥3 months before screening	104 weeks
HARMONY 5	NCT00839527	Subcutaneous injection of albiglutide 30 mg/week	271	115	Type 2 diabetes mellitus on metformin and a sulfonylurea and BMI 20−45 kg/m^2^ and HbA_1c_ 7.0%−10.0%	156 weeks
STEP‐HFpEF DM	NCT04916470	Once‐weekly subcutaneous semaglutide escalated from 0.25 to 2.4 mg added to baseline antihyperglycemic medications	310	306	Heart failure with LVEF ≥ 45% and BMI ≥ 30 kg/m^2^ and type 2 diabetes	52 weeks
O'Neil 2018	NCT02453711	0.05–0.4 mg daily subcutaneous semaglutide or 3.0 mg liraglutide once daily	821	136	Individuals with BMI ≥ 30 kg/m^2^ without diabetes	52 weeks
PIONEER 8	NCT03021187	Oral semaglutide 3, 7, or 14 mg	546	184	Type 2 diabetic patients (HbA_1c_ 7%−9.5%) on insulin with or without metformin use	52 weeks
SCALE Obesity and Prediabetes	NCT01272219	Once‐daily 3.0 mg subcutaneous liraglutide	2487	1244	Overweight with dyslipidemia or hypertension/obese patients without type 2 diabetes	56 weeks
SCALE Diabetes	NCT01272232	Once‐daily subcutaneous injection of 1.8 or 3.0 mg of liraglutide	634	212	Type 2 diabetics with BMI ≥ 27 kg/m^2^ on 0−3 hypoglycemic agents	56 weeks
SCALE Maintenance	NCT00781937	Once‐daily subcutaneous injection of 3.0 mg of liraglutide	212	210	BMI ≥ 27 with comorbidities or ≥30 kg/m^2^ who lost 5% or more of body weight during a low‐calorie diet run‐in	56 weeks

Abbreviations: BMI, body mass index; CKD, chronic kidney disease; HFpEF, heart failure with preserved ejection fraction; HFrEF, heart failure with reduced ejection fraction; MI, myocardial infarction; T2DM, type 2 diabetes mellitus.

### 3.2 Risk of Bias

**Figure 2:**
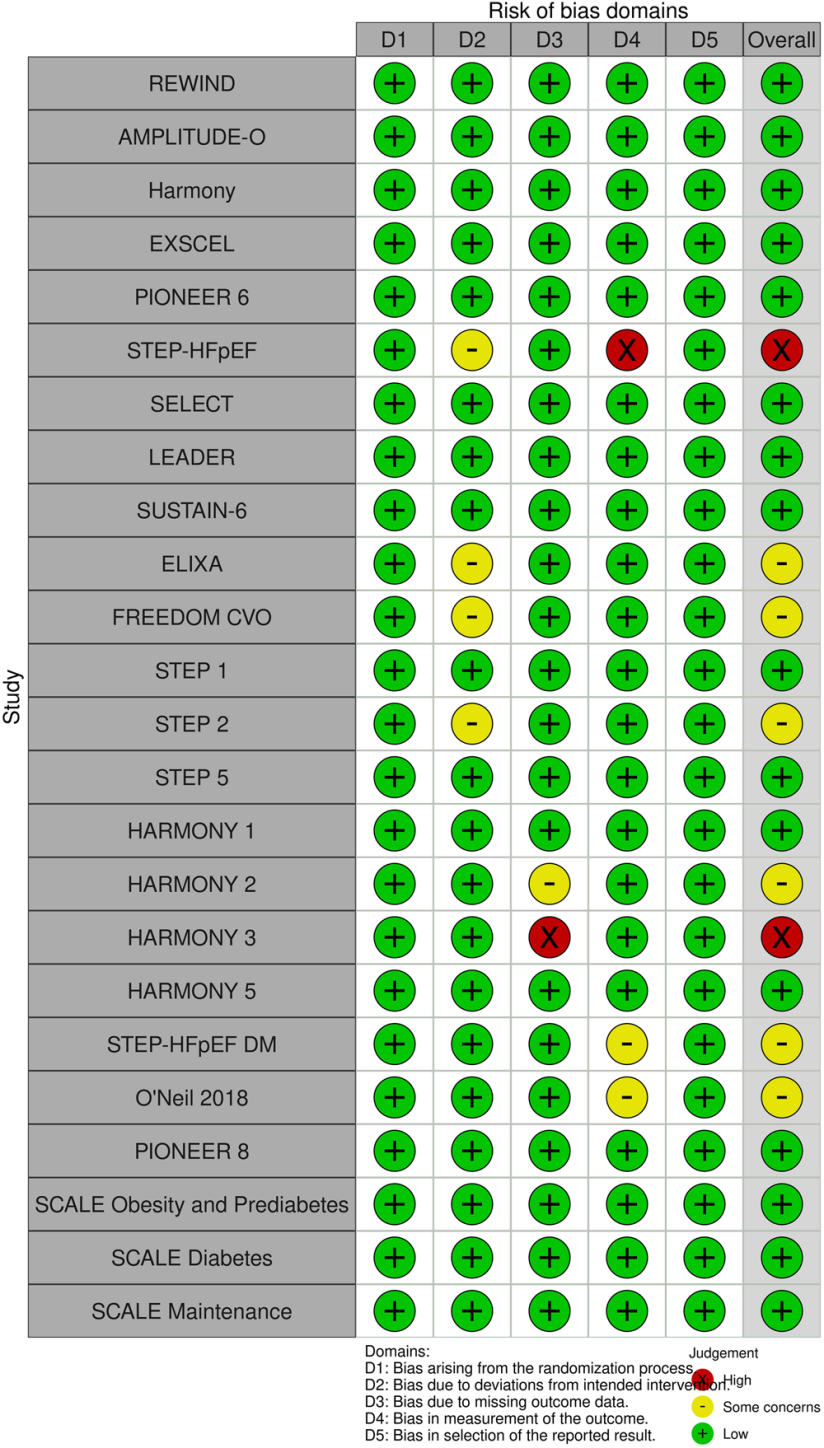
Traffic light of the risk of bias assessment (REWIND: the Researching Cardiovascular Events with a Weekly Incretin in Diabetes, AMPLITUDE‐O, Harmony, EXSCEL: the Exenatide Study of Cardiovascular Event Lowering, PIONEER 6: Peptide Innovation for Early Diabetes Treatment 6, STEP‐HFpEF: Semaglutide in Patients with Heart Failure with Preserved Ejection Fraction and Obesity, SELECT: the Semaglutide Effects on Cardiovascular Outcomes in People with Overweight or Obesity, LEADER: Liraglutide Effect and Action in Diabetes: Evaluation of Cardiovascular Outcome Results, SUSTAIN‐6: Trial to Evaluate Cardiovascular and Other Long‐term Outcomes With Semaglutide in Subjects With Type 2 Diabetes, ELIXA: Evaluation of Lixisenatide in Acute Coronary Syndrome, STEP: Semaglutide Treatment Effect in People with Obesity, SCALE: Satiety and Clinical Adiposity—Liraglutide Evidence in Nondiabetic and Diabetic Individuals).

Quality appraisal of the included trials showed an overall low risk of bias for 16 trials [3, 8, 16, 17, 19, 20, 21, 22, 25, 29, 30, 31, 32, 33, 34, 35] and six trials [14, 18, 24, 26, 27, 28] were rated as having some concerns. Also, two trials [15, 23] showed an overall high risk of bias mainly driven from measurement of the outcome and deviation from the intended intervention and also missing outcome data. All of the included trials were at low risk of potential bias arising from randomization, allocation concealment, and selective reporting of the results. A total of four trials [14, 15, 18, 24] were assumed to have some or a high level of bias due to deviations from the intended intervention as there was a high level of nonadherence (discontinuation mainly due to adverse gastrointestinal side effects), with no sensitivity analysis limited to per‐protocol population confirming the overall results, which could have potentially affected the final results (Figure 2).

### 3.3 GLP‐1 Agonists and Major Adverse Clinical Events

**Figure 3:**
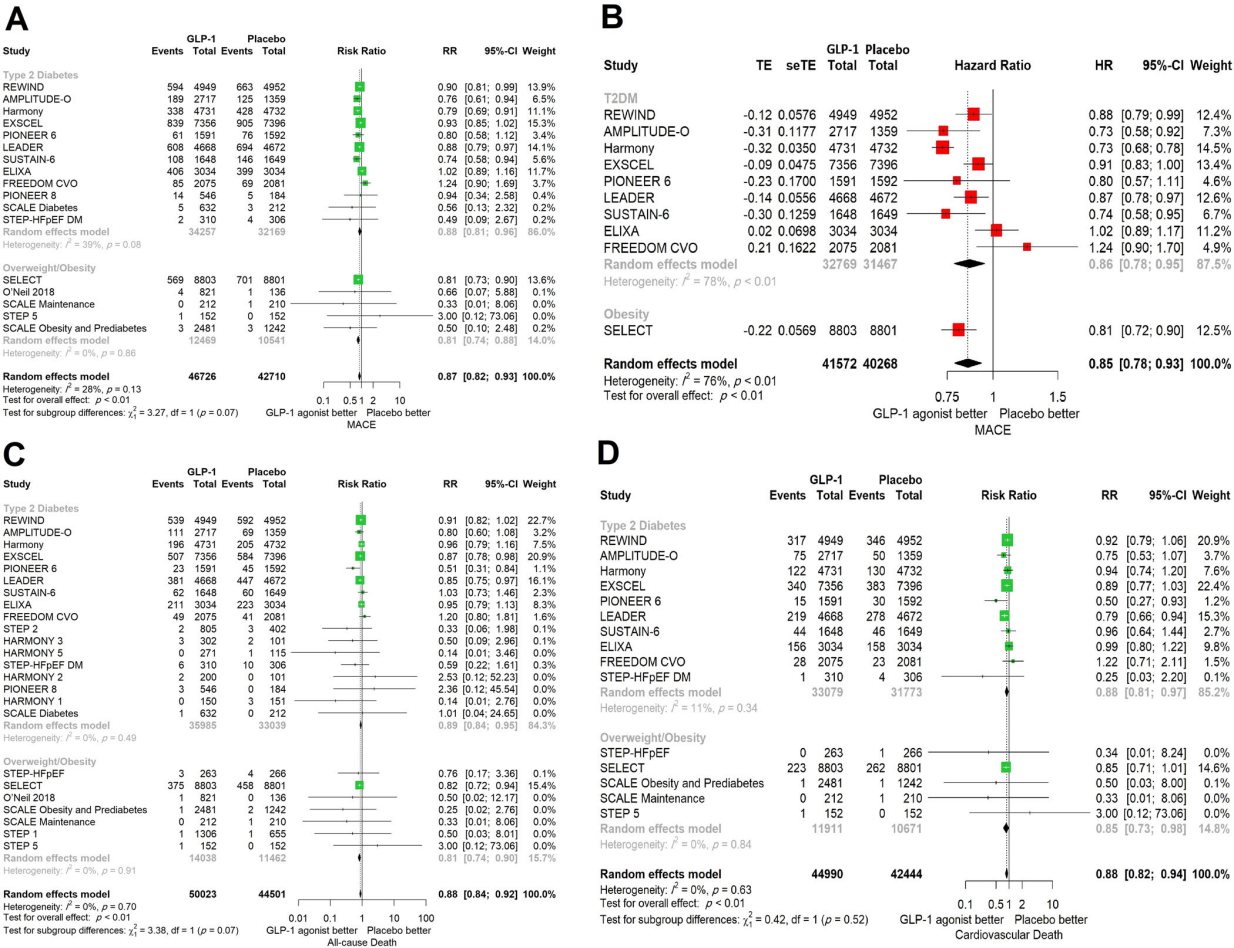
Comparison between GLP‐1 receptor agonists and placebo stratified by comorbidity in terms of (A) MACE (RR), (B) MACE (HR), (C) all‐cause death, and (D) cardiovascular death. CI, confidence interval; GLP‐1, glucagon‐like peptide‐1; HR, hazard ratio; MACE, major adverse cardiovascular events; RR, relative risk.

**Figure 4:**
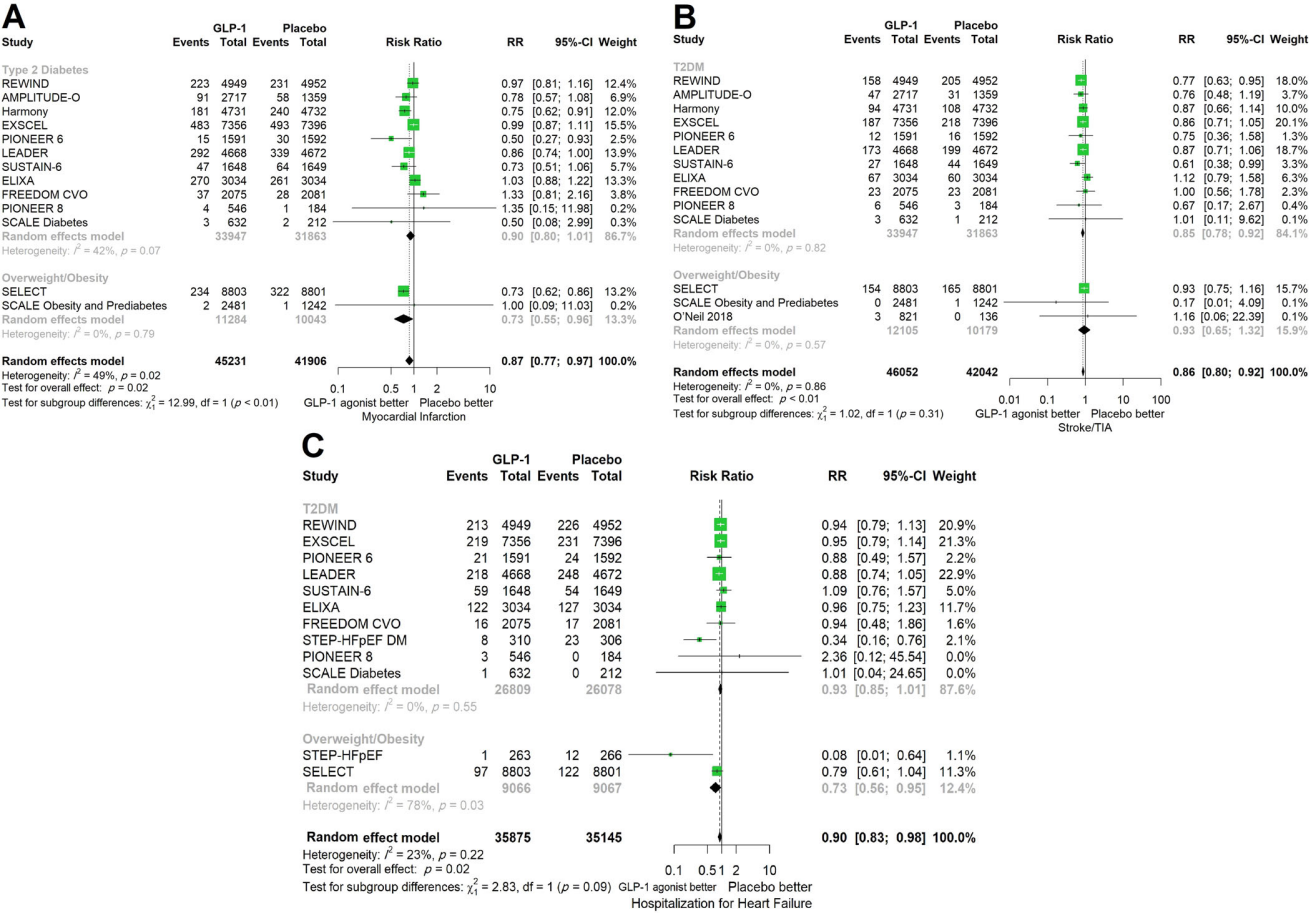
Comparison between GLP‐1 receptor agonists and placebo stratified by comorbidity in terms of (A) myocardial infarction, (B) stroke, and (C) hospitalization for heart failure. CI, confidence interval; GLP‐1, glucagon‐like peptide‐1; RR, relative risk.

In our analysis, patients on GLP‐1 RAs had a statistically significant decrease in RR of MACE in both subgroups of patients with type 2 diabetes (RR 0.88, 95% CI 0.81−0.96) and overweight/obese patients (RR 0.81, 95% CI 0.74−0.88) compared with placebo (overall population: RR 0.87, 95% CI 0.82−0.93, *I* ^2^ = 28%, *p* = 0.0005) (Figure 3A). Taking GLP‐1 RAs was associated with lower HR of MACE compared with placebo (HR 0.85, 95% CI 0.78−0.93, *p* = 0.0002) in both diabetic patients (HR 0.86, 95% CI 0.78−0.95, *p* < 0.01) and overweight/obese patients (HR 0.81, 95% CI 0.72−0.90), with a substantial level of heterogeneity among studies on diabetic patients (*I* ^2^ = 78%, *p* < 0.01) (Figure 3B). There was a 12% risk reduction in the GLP‐1 group in terms of all‐cause death across the whole population (*n* = 94 524, RR 0.88, 95% CI 0.84−0.92, *p* < 0.0001). This association was observed in patients with diabetes (*n* = 69 024, RR 0.89, 95% CI 0.84−0.95) and overweight/obese patients (*n* = 25 500, RR 0.81, 95% CI 0.74−0.90) (Figure 3C). Comparison of the GLP‐1 group with controls demonstrated a 12% risk reduction in cardiovascular‐related death (*n* = 87 434, RR 0.88, 95% CI 0.82−0.94, *p* = 0.0011) and both subgroups of type 2 diabetics (*n* = 64 852, RR 0.88, 95% CI 0.81−0.97), and overweight/obese patients without diabetes (*n* = 22 582, RR 0.85, 95% CI 0.73−0.98) had statistically significantly reduced risk of cardiovascular mortality (Figure 3D). The overall RR of MI (RR 0.87, 95% CI 0.77−0.97, *p* = 0.0190) (Figure 4A) and stroke (RR 0.86, 95% CI 0.80−0.92, *p* = 0.0006) (Figure 4B) was significantly lower in the GLP‐1 group than the placebo group. In the diabetic subgroup, GLP‐1 agonists decreased the RR of developing stroke (RR 0.85, 95% CI 0.78−0.92) but the association for MI was marginally nonsignificant (RR 0.90, 95% CI 0.80−1.01, *I* ^2^ = 42%). In overweight/obese patients without diabetes, taking GLP‐1 RAs could reduce the risk of MI by 27% (RR 0.73, 95% CI 0.55−0.96), but there was no significant change in the risk of stroke (RR 0.93, 95% CI 0.65−1.32). The risk of hospitalization for heart failure was decreased by 10% in the GLP‐1 RA group compared with the placebo group (RR 0.90, 95% CI 0.83−0.98, *p* = 0.02). This association was statistically significant in the subgroup of overweight/obese patients (RR 0.73, 95% CI 0.56−0.95) but not in diabetic patients (RR 0.93, 95% CI 0.85−1.01, *I* ^2^ = 0%), although no significant difference was observed between subgroups (*p* = 0.09) (Figure 4C). Sensitivity analysis showed no significant change in results on omitting studies one at a time.

## 4 Discussion

The relationship between GLP‐1 RAs and cardiovascular health has transformed the approach to managing not only diabetes but also broader cardiovascular conditions. GLP‐1 RAs were initially lauded for glycemic control, but their potential in averting MACE has captured the medical community's attention. Multiple studies consistently showed reduced cardiovascular risk among patients receiving these agents. Significant reductions in MACE, MIs, strokes, and cardiovascular mortality underscore the potential cardioprotective effects of GLP‐1 RAs. Beyond glucose regulation, these agonists exert vasodilatory effects, reduce inflammation, improve endothelial function, and potentially stabilize plaques—factors crucial in averting adverse cardiovascular events [36, 37, 38]. This multifaceted action suggests a more extensive influence on the cardiovascular system than initially thought. Leveraging GLP‐1 receptor agonists in managing cardiovascular risk factors among nondiabetic individuals challenges traditional paradigms. Incorporating these agents into existing treatment algorithms for a wider patient population sparks discussions on optimal therapeutic strategies.

In the present analysis of 94 547 participants, the following key findings should be noted: (1) Overall, use of GLP‐1 RAs in patients with diabetes and overweight/obese patients without diabetes showed significant risk reduction compared with placebo in terms of MACE, all‐cause and cardiovascular death, MI, stroke/TIA, and hospitalization for heart failure; (2) both diabetic patients and overweight/obese patients without diabetes demonstrated improved outcomes regarding MACE, all‐cause death, and cardiovascular mortality and no statistically significant difference was noted between the subgroups; (3) the incidence of stroke was decreased in the diabetic subgroup but not in the overweight participants without diabetes; and (4) type 2 diabetic patients had marginally nonsignificant risk reduction in MI and hospitalization for heart failure, whereas overweight participants without diabetes showed significant results, although the results were mostly derived from one single trial.

The study population of this meta‐analysis included either patients diagnosed with type 2 diabetes or overweight/obese patients without diabetes. Almost all participants were either diagnosed with previous episodes of CVDs or had high cardiovascular risk based on the trial inclusion criteria. The remarkable novelty of our analysis is that this is the first meta‐analysis to assess cardiovascular outcomes in patients based on their baseline comorbidity (with and without diabetes). Our primary endpoint of interest (MACE) showed benefits toward better outcomes in patients taking the GLP‐1 RAs irrespective of the baseline condition. Our analysis demonstrated that the MACE was reduced by 15% among patients with diabetes or overweight/obese patients without diabetes (HR 0.85, 95% CI 0.78−0.93). It is noteworthy that a smaller number of trials studied the effects of GLP‐1 RAs in overweight/obese patients and nondiabetic individuals as trials investigating the impact of these agents on nondiabetic patients are starting to emerge. The recently published SELECT trial was the first large‐scale randomized study comparing the cardiovascular outcomes of GLP‐1 agonists with placebo as its primary endpoint of interest in patients who were overweight/obese but not diabetic [8]. The important finding that the present analysis highlighted is that the efficacy of GLP‐1 agonists in secondary prevention of adverse cardiovascular outcomes in patients with underlying overweight/obesity but no diabetes is generally similar and even has a greater magnitude of effect compared to CVOTs of patients with diabetes and established cardiovascular conditions (RR = 0.81 vs. 0.88). Previous meta‐analyses on the cardiovascular outcomes of GLP‐1 RAs have limited their studied population to the subset of patients with diabetes. A previous meta‐analysis of eight trials and 60 080 patients showed a significant reduction in the risk of MACE, mortality, infarction, and stroke [36]. Herein, we extended the included population to patients with diabetes and also overweight/obese individuals without diabetes. Our results showing a significant improvement in cardiovascular outcomes regardless of the underlying diabetes are of great importance and should motivate future randomized trials to further assess the cardiovascular efficacy of GLP‐1 agonists in patients with cardiovascular conditions without diabetes.

The majority of the Phase 3 trials on the cardiovascular outcomes of GLP‐1 RAs recruited participants with either established CVD (usually defined as a previous episode of MI, stroke, revascularization, or coexisting peripheral vascular disease) or those with a high cardiovascular risk. Long‐acting GLP‐1 RAs have been proven to be effective in reducing long‐term MACE in some trials in which a high proportion (> 80%) of patients (e.g., SUSTAIN‐6 [16] and LEADER [19]) or the whole eligible population (Harmony Outcomes [22]) had established CVD. Studies on lower‐risk populations such as the REWIND trial, in which 31% of participants had coexisting cardiovascular conditions, also showed reduced risk of MACE after 6 years of dulaglutide use [20]. Consistent with the results published from most of the trials on diabetic patients, our analysis also showed the cardiovascular efficacy of GLP‐1 RAs in the subgroup of patients with type 2 diabetes.

Randomized‐controlled trials studying the cardiovascular effects of GLP‐1 RAs in nondiabetics are starting to emerge. The target population of these trials has usually included overweight/obese patients and those either at high risk for cardiovascular events or with an established cardiovascular condition. The SELECT trial evaluated the cardiovascular outcomes of subcutaneous semaglutide in overweight/obese patients with established CVD and no diabetes. After 4 years of follow‐up, semaglutide injection was superior to placebo regarding MACE, heart failure events, and all‐cause death [8]. The STEP‐HFpEF was another trial that enrolled more than 500 patients with a diagnosis of heart failure with ejection fraction ≥45% and BMI ≥ 30 kg/m^2^ who were assigned to receive a weekly injection of semaglutide or placebo. Although heart failure events were higher in the placebo group, the rate of all‐cause and cardiovascular‐related mortality was similar among the groups. The relatively small duration of follow‐up (1 year) and also the small sample size may have contributed to these nonsignificant clinical outcomes. In addition, MACEs were not among the primary outcomes of interest, and this trial was underpowered to accurately compare these outcomes across the groups that were studied [15]. Several other trials have also reported adjudicated cardiovascular events as the secondary outcomes in nondiabetic populations taking GLP‐1 RAs [28, 33, 34]. A recently published meta‐analysis on the efficacy and safety of GLP‐1 RAs in overweight or obese patients with no diabetes has shown better MACE but similar stroke and cardiovascular death in the experimental arm compared with placebo [39]. It should be noted that the mentioned study included trials on tirzepatide, which is a dual GIP/GLP‐1 agonist, and also trials with a follow‐up duration of less than 1 year, which may have limited the relevant impact of these agents on cardiovascular events. The results of the present meta‐analysis showed that GLP‐1 RAs have cardioprotective effects in overweight or obese patients without diabetes and more Phase 3 trials studying on nondiabetic patients are warranted to confirm our results.

The duration of the action of GLP‐1 RAs is another determining factor in the clinical efficacy of these medications as short‐acting agents have a half‐life of 2−3 h, limiting the therapeutic range to only a few hours per day [40]. This was further demonstrated in the results of the ELIXA trial evaluating the cardiovascular effects of lixisenatide, a short‐acting GLP‐1 RA, in patients with type 2 diabetes and a recent episode of acute coronary syndrome. The addition of lixisenatide to the standard care did not show any superiority compared with placebo in terms of any of the cardiovascular‐related outcomes [18]. It is also hypothesized that there may be a potential delay between the initiation of therapy and the appearance of cardiovascular benefits, and thus, future studies should have a longer duration of follow‐up. A non‐inferiority trial was designed to investigate the outcomes of ITCA 650, a continuous sustained‐release form of exenatide, using an osmotic mini‐pump to boost the adherence of the drug. The trial failed to show any superiority over placebo, although the short follow‐up duration and the study design focused on non‐inferiority may have hindered the ability to demonstrate any associations, requiring further confirmation [14]. It can be concluded that prolonging the trial duration and also using long‐acting GLP‐1 RAs are among the determining factors influencing the cardiovascular efficacy of these agents in the studies and choosing an optimal trial duration and long‐acting agents may result in better cardioprotective effects in the GLP‐1 RA trials.

Our analysis had certain limitations. The trials on patients with no diabetes were limited compared with studies on diabetic patients. Other than the results of the SELECT trial, studies on patients without diabetes showed inconclusive results as they were underpowered for clinical outcomes and sample size. Also, substantial heterogeneity is observed in the results for MACE. This variability may be attributed to several factors including differences in the duration of the effect of medications (short‐ vs. long‐acting), duration of follow‐up, targeted population, and study design. We should mention that we performed sensitivity analysis in this regard, and no change was observed in the results.

## 5 Conclusion

In conclusion, our analysis showed that GLP‐1 RAs are superior to placebo regarding MACE, all‐cause and cardiovascular mortality, MI, stroke, and hospitalization for heart failure. GLP‐1 agonists showed a similar magnitude of effect in overweight/obese patients without diabetes compared to patients with diabetes in terms of MACE, all‐cause death, and cardiovascular‐related death. The cardiovascular benefits of GLP‐1 agonists may not be limited to patients with diabetes and this group of medications may be applied in a broader population including overweight/obese patients.

## Conflicts of Interest

The authors declare no conflicts of interest.

## Data Availability

The data underlying this article will be shared on reasonable request to the corresponding author.
